# Continued Susceptibility of the *w*Mel *Wolbachia* Infection in *Aedes aegypti* to Heat Stress Following Field Deployment and Selection

**DOI:** 10.3390/insects9030078

**Published:** 2018-06-30

**Authors:** Perran A. Ross, Ary A. Hoffmann

**Affiliations:** Pest and Environmental Adaptation Research Group, School of BioSciences, Bio21 Institute, The University of Melbourne, Victoria 3010, Australia; ary@unimelb.edu.au

**Keywords:** *Aedes aegypti*, *Wolbachia*, heat stress, experimental evolution, cytoplasmic incompatibility

## Abstract

*Aedes aegypti* mosquitoes infected with the *w*Mel strain of *Wolbachia* are being deployed to control the spread of arboviruses around the world through blockage of viral transmission. Blockage by *Wolbachia* in some scenarios may be affected by the susceptibility of *w*Mel to cyclical heat stress during mosquito larval development. We therefore evaluated the potential to generate a heat-resistant strain of *w*Mel in *Ae. aegypti* through artificial laboratory selection and through exposure to field temperatures across multiple generations. To generate an artificially selected strain, *w*Mel-infected females reared under cyclical heat stress were crossed to *w*Mel-infected males reared at 26 °C. The low proportion of larvae that hatched founded the next generation, and this process was repeated for eight generations. The *w*Mel heat-selected strain (*w*Mel-HS) was similar to *w*Mel (unselected) in its ability to induce cytoplasmic incompatibility and restore compatibility when larvae were reared under cyclical heat stress, but *w*Mel-HS adults exhibited reduced *Wolbachia* densities at 26 °C. To investigate the effects of field exposure, we compared the response of *w*Mel-infected *Ae. aegypti* collected from Cairns, Australia where the infection has been established for seven years, to a *w*Mel-infected population maintained in the laboratory for approximately 60 generations. Field and laboratory strains of *w*Mel did not differ in their response to cyclical heat stress or in their phenotypic effects at 26 °C. The capacity for the *w*Mel infection in *Ae. aegypti* to adapt to high temperatures therefore appears limited, and alternative strains may need to be considered for deployment in environments where high temperatures are regularly experienced in mosquito breeding sites.

## 1. Introduction

The open field release of *Wolbachia*-infected mosquitoes is a promising approach to control the spread of arboviruses and has gained considerable attention in recent years [1,2,3]. *Wolbachia* infections can protect their hosts against pathogens, and this feature has been exploited to generate strains of mosquitoes with a reduced ability to transmit arboviruses [4,5]. The principal vector of dengue, the *Aedes aegypti* mosquito, has now been infected experimentally with several strains of *Wolbachia* originating from other insects [4,6,7,8,9,10,11]. The majority of these *Wolbachia* strains induce cytoplasmic incompatibility, which greatly reduces or eliminates the production of viable offspring by uninfected females that mate with *Wolbachia*-infected males. This feature combined with *Wolbachia*-induced viral suppression has prompted the deployment of *Ae. aegypti* with artificial *Wolbachia* infections into the field in areas where dengue and other arboviruses are endemic. Efforts such as the World Mosquito Program (formerly Eliminate Dengue) (www.worldmosquitoprogram.org) and Wolbachia Malaysia (http://repository.imr.gov.my/wolbachia) aim to replace natural populations of *Ae. aegypti* mosquitoes with *Wolbachia*-infected mosquitoes that are less receptive to arbovirus transmission, while other groups aim to utilize cytoplasmic incompatibility to suppress mosquito populations through the release of only males (https://blog.debug.com; http://www.nea.gov.sg/public-health/environmental-public-health-research/wolbachia-technology/project-wolbachia-singapore).

The suitability of a given *Wolbachia* strain for population replacement programs will depend largely on its effects on mosquito fitness, the fidelity of maternal transmission, and cytoplasmic incompatibility and its ability to block arboviruses in field populations [12]. The *w*Mel strain originating from *Drosophila melanogaster* was chosen for its relative lack of fitness costs and its ability to induce complete cytoplasmic incompatibility, inhibit dengue virus replication, and be transmitted maternally with perfect fidelity in the laboratory [8]. *w*Mel-infected *Ae. aegypti* have now established in multiple field populations in Queensland, Australia following field deployment [1,2]. Infected mosquitoes persisted at high frequencies in release zones years after releases ceased [2,13] and have retained their ability to inhibit dengue virus replication [14] and induce cytoplasmic incompatibility [13]. In recent years, *w*Mel-infected *Ae. aegypti* have been released successfully in further trials in Asia and South America (http://www.eliminatedengue.com/project).

The success of these trials demonstrates the utility of the *w*Mel strain to invade natural populations, and infected mosquitoes isolated from natural populations can block virus transmission [15]. However, deployment of the *w*Mel strain may be limited by certain environmental conditions. Fitness costs of *w*Mel infection can arise in competitive environments [16,17], which could reduce invasiveness, though some studies observe fitness benefits or find no effect of infection [18,19,20]. The extent to which the *w*Mel strain will reduce dengue transmission in the field may also be affected by environmental conditions. Early studies demonstrated a strong viral inhibition phenotype by *w*Mel [8], but there is variability in the ability of *Wolbachia* to suppress native viruses in mosquitoes fed on the blood of dengue patients [15,21]. Transmission blocking by the *w*Mel infection may also vary with virus load [22]. Alternative *Wolbachia* infections such as *w*MelPop and *w*Au possess superior viral blockage to *w*Mel but are also costlier to host fitness [11,21,23].

Temperature conditions can have considerable impacts on insect symbionts [24] and affect the ability of *Wolbachia* infections to invade *Ae. aegypti* populations in the field and reduce viral transmission. Heat stress can disrupt and deform the cellular membranes of *Wolbachia*, altering morphology and leading to degeneration [25,26]. In *Ae. aegypti*, cyclical heat stress reduces the ability of *w*Mel to induce cytoplasmic incompatibility, lowers its density in the mosquito and can even eliminate the infection from subsequent generations [11,27,28]. A low level of incomplete maternal transmission of *w*Mel was recently reported in an Australian field population [29], which could be explained by heat stress experienced in mosquito larval habitats. *w*Mel in its native *D. melanogaster* host is also temperature-sensitive, where its abundance relative to other microbes is greatly reduced when flies develop at 31 °C [30]. The thermal sensitivity of *Wolbachia* infections are variable and can differ considerably between host species [24]. In *Ae. aegypti*, the *w*AlbB infection is more stable than *w*Mel and *w*MelPop at high temperatures; *Wolbachia* strains in the same host can therefore also vary in their response to heat stress [28].

*Wolbachia* and other symbionts can exhibit a large amount of genetic diversity within a single insect host [31], and with this variation brings the potential for selection [32]. There are now several demonstrations of *Wolbachia* infections rapidly adapting to changes in environment, including cases where virulence has increased [33] or decreased [34,35,36] following experimental transfer to new hosts. *D. melanogaster* populations selected for increased survival after viral challenge led to the fixation of more protective *Wolbachia* variants [37]. Selection for *Wolbachia* variants with increased Octomom copy numbers increased the density and virulence of *Wolbachia* [38]. Parthenogenic *Trichogramma cordubensis* exhibit variability in their response to high temperature conditions, and selection under a constant 30 °C increased the strength of parthenogenesis under heat stress [39]. In *D. melanogaster*, frequencies of different *Wolbachia* variants changed dramatically when flies were maintained at different temperatures [40], and there is evidence of selection on the *w*Mel strain for genes encoding heat shock proteins [41]. These studies demonstrate that *Wolbachia* infections can evolve in response to a changing environment and raise the possibility that experimental evolution or direct selection could therefore be used as a tool to select *Wolbachia* infections in *Ae. aegypti* mosquitoes for desirable attributes, improving their utility for arbovirus control applications.

In this study, we subjected *Ae. aegypti* infected with *w*Mel to cyclical heat stress over successive generations in an attempt to improve the thermal tolerance of this *Wolbachia* infection. We also tested whether variable field temperatures had led to any shift in the thermal responses of *Wolbachia* by comparing a laboratory line exposed to a constant temperature with mosquitoes derived from the field population where the original releases of *w*Mel had taken place seven years ago [1].

## 2. Materials and Methods

### 2.1. Ethics Statement

Blood feeding on human subjects was approved by the University of Melbourne Human Ethics Committee (approval 0723847). All volunteers provided informed written consent.

### 2.2. Mosquito Strains and Colony Maintenance

*Ae. aegypti* mosquitoes with the *w*Mel infection were collected in 2013 from locations near Cairns, Australia where *w*Mel had successfully established [1]. Uninfected *Ae. aegypti* were collected in 2016 from locations where *w*Mel-infected mosquitoes had not been released. The *w*AlbB infection was used for comparisons in some cytoplasmic incompatibility experiments and is described in Xi, Khoo, and Dobson [6] and Axford, et al. [42]. *Wolbachia*-infected mosquitoes were crossed to uninfected mosquitoes for at least three consecutive generations to ensure similar genetic backgrounds before use in experiments. Colonies were maintained in the laboratory at 26 ± 1 °C according to Ross, et al. [43]. *w*Mel-infected *Ae. aegypti* were also collected from Yorkeys Knob, Australia in January 2018, where the *w*Mel infection is established in the population following initial releases there seven years ago [1]. At the end of the study, the *w*Mel infection from mosquitoes collected in 2013 had been maintained in the laboratory for approximately 60 generations, while the heat selected *w*Mel population had been through eight generations of selection. These three populations were compared in a later set of experiments.

### 2.3. Selection Regime

We subjected infected *Ae. aegypti* to cyclical heat stress over successive generations. First instar *w*Mel-infected larvae were placed in incubators (PG50 Plant Growth Chambers, Labec Laboratory Equipment, Marrickville, NSW, Australia) set to cycle diurnally between 26 °C and 37 °C, with 12 h at each temperature, according to Ross, et al. [28]. Up to four trays with 100 larvae each in 500 mL of RO (reverse osmosis) water were reared under these conditions each generation. Data loggers (Thermochron; 1-Wire, iButton.com, Dallas Semiconductors, Sunnyvale, CA, USA) in zip-lock bags were placed in each tray to monitor temperature. Larvae were provided with TetraMin^®^ tropical fish food tablets (Tetra, Melle, Germany) *ad libitum* until reaching pupation. Females were separated from males at the pupal stage and returned to 26 °C for adult emergence, and males were discarded. Adult females were held at 26 °C for three days before being crossed to *w*Mel-infected males (unselected) reared at 26 °C. Females were blood-fed on a single human volunteer, and all eggs were hatched in 3 L trays of RO water with a few grains of yeast. The resulting progeny founded the next generation, and the selection regime was repeated. Subsequent gonotrophic cycles were initiated if more eggs were needed.

Crosses with *w*Mel-infected males reared at 26 °C were chosen to produce low hatch proportions, resulting from partial cytoplasmic incompatibility. This increased the intensity of selection as only females that restored compatibility when reared under heat stress would contribute to the next generation, eliminating any individuals that lost their *Wolbachia* infection. Hatch proportions also provided an indicator of the response to selection; high hatch proportions in subsequent generations would suggest an improvement in heat tolerance.

After each generation of selection, a subset of progeny was reared at 26 °C as a backup in case the *Wolbachia* infection from the selected line was lost during the next selection event. After two generations of selection, all progeny were reared at 26 °C to increase the population size as hatch proportions were low (Table 1). After five generations of selection, a subset of progeny was reared at 26 °C for two generations, where females from the selected lines were crossed to *w*Mel (unselected) males. The resulting progeny were then used for cytoplasmic incompatibility experiments and measured for *Wolbachia* density. Egg hatch proportions were determined by observation under a dissecting microscope and were recorded for the first eight generations. Hatched eggs had a clearly detached cap and no larva inside. We continued selection for three further generations and performed an additional set of experiments that included a *w*Mel infection that had been maintained in the laboratory at 26 °C for approximately 60 generations (*w*Mel-lab), and a *w*Mel infection that had been in the field for seven years (*w*Mel-field).

### 2.4. Cytoplasmic Incompatibility and Restoration of Compatibility

After five generations of selection followed by two relaxed generations, we tested the ability of the selected *w*Mel strain (from now on referred to as *w*Mel-HS) to induce cytoplasmic incompatibility in crosses with uninfected females and restore compatibility in crosses with *w*Mel-infected males. Larvae from the *w*Mel (unselected), *w*Mel-HS, and *w*AlbB lines were reared at a constant 26 °C or in incubators set to cycle diurnally between 26 °C and 37 °C, with 12 h at each temperature. Uninfected larvae were reared at 26 °C only for crosses with infected males. We generated two heat stress treatments which differed in the maximum daily temperature reached by 1 °C by utilizing variation within the incubator. Trays were reared on different shelves of the same incubator, reaching average maximum daily temperatures of 36.5 °C and 37.5 °C, respectively (Figure S1A). Variation between containers on the same shelf was negligible, where they were cycled in their position daily. After five days, larvae from the heat stressed treatments were returned to 26 °C for pupation and adult emergence. Four trays with 100 larvae each were reared for each line and each temperature treatment. Adults from each sex, temperature, and infection status were maintained separately at 26 °C for three days. We then set up nine crosses to test the ability of males from each infected line under each rearing condition to induce cytoplasmic incompatibility with uninfected females. We set up a further nine crosses with females from each infected line under each rearing condition to test their ability to restore compatibility with infected males. For crosses with females from the *w*Mel and *w*Mel-HS lines, males from the unselected *w*Mel line were used. For crosses with *w*AlbB females, *w*AlbB males were used. We added 25 males and 25 females to 1.5 L cages for each cross and allowed two days to mate. After blood feeding, 20 females from each cross were isolated in 70 mL cups containing 20 mL of larval rearing water and that were lined with sandpaper for oviposition. Eggs were collected from each cup four days after blood feeding, partially dried, then hatched four days after collection. After one week, females were blood fed again, and eggs were collected and hatched from a second gonotrophic cycle.

After eight generations of selection followed by two relaxed generations, we conducted a similar set of experiments where we included a *w*Mel-infected population maintained in the laboratory for approximately 60 generations (*w*Mel-lab) and a population collected from the field where the *w*Mel infection had been present for seven years (*w*Mel-field). *w*Mel-field was maintained in the laboratory for two generations before experiments began. In this experiment, we reared larvae at 26 °C or under heat stress treatments reaching average maximum daily temperatures of 36 °C and 37 °C (Figure S1B). Methods for rearing and crosses were otherwise identical to the first experiment, except we tested egg hatch proportions from a single gonotrophic cycle only.

### 2.5. Wolbachia Quantification

A subset of adults from both experiments were stored in absolute ethanol within 24 h of emergence and measured for their *Wolbachia* density. DNA was extracted from twenty adults from each sex and infection, and *Wolbachia* density was estimated with qPCR using previously described methods [28,44].

### 2.6. Statistical Analysis

All data were analysed in SPSS statistics version 24.0 for Windows (SPSS Inc., Chicago, IL, USA). Egg hatch proportions and *Wolbachia* densities were compared with nonparametric Kruskal-Wallis and Mann-Whitney U tests, as data were not normally distributed according to Shapiro-Wilk tests. We used a general linear model to test for an effect of female age on egg hatch proportions during the selection experiment.

## 3. Results

### 3.1. Egg Hatch Proportions during Selection

We monitored egg hatch proportions for the first eight generations as an indication of the selection response. Overall hatch proportions during the first generation of selection were intermediate and varied considerably between individual females, but from the second generation of selection onwards they fell consistently below 4% (Table 1). The *w*Mel infection was lost in the selected line after the seventh generation of heat stress. No eggs hatched, and *Wolbachia* was not detected by qPCR in a sample of 30 individuals from the population. However, selection continued for a total of eight generations with a backup line.

**Figure 1:**
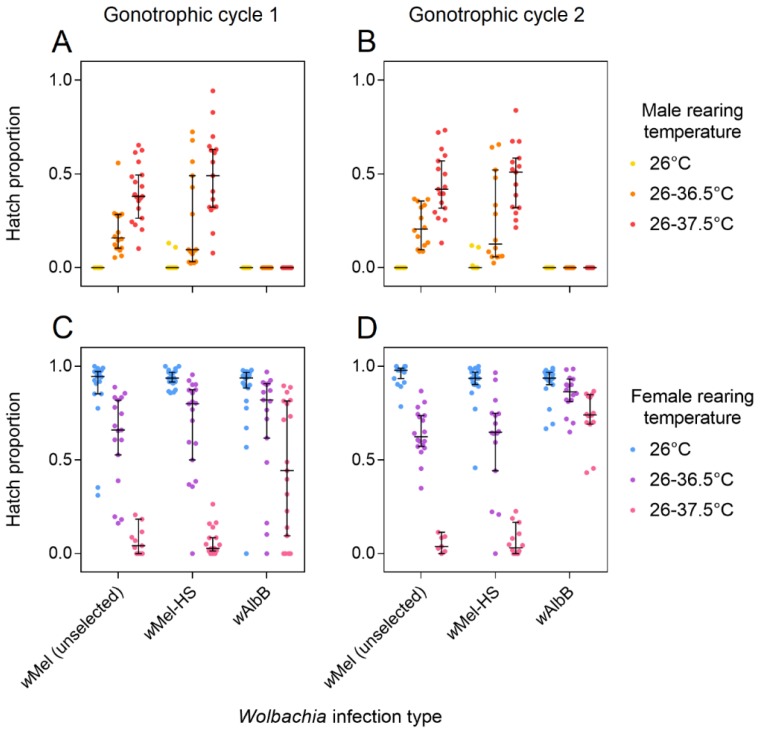
The ability of *w*Mel to induce cytoplasmic incompatibility and restore compatibility under heat stress after five generations of selection. Males (**A**,**B**) and females (**C**,**D**) from the *w*Mel (unselected), *w*Mel-HS (*w*Mel heat-selected strain) and *w*AlbB lines were reared at a constant 26 °C, or held in incubators that cycled diurnally between 26 °C and 36.5 °C or 26 °C and 37.5 °C. (**A**,**B**) Males reared at each temperature were crossed to uninfected females to test their ability to induce cytoplasmic incompatibility. Egg hatch proportions were measured over both the first (**A**) and second (**B**) gonotrophic cycles. (**C**,**D**) Females reared at each temperature were crossed to infected males reared at 26 °C to test their ability to restore compatibility. Egg hatch proportions were measured over both the first (**C**) and second (**D**) gonotrophic cycles. Black bars indicate median egg hatch proportions with 95% confidence intervals.

Egg hatch proportions of females from the first generation of heat stress increased dramatically in subsequent gonotrophic cycles (Kruskal-Wallis: χ^2^ = 26.616, df = 2, *p* < 0.001, Figure S2A). This restoration of compatibility over time indicates a recovery of *Wolbachia* within the female. However, we observed no such recovery in subsequent selection generations when initial hatch proportions were much lower (Figure S2B), or during the first cytoplasmic incompatibility experiment, except for the *w*AlbB infection (Figure 1). Egg hatch proportions of females from selection generations 2–7 were consistently low and did not change with female age according to a general linear model (F_1,65_ = 0.629, *p* = 0.841, Figure S2B). We also tested if hatch proportions increased when heat stressed females were aged before mating with *Wolbachia*-infected males, but found no improvement with female age (Figure S3).

### 3.2. Cytoplasmic Incompatibility and Restoration of Compatibility Following Selection

After five generations of selection, we tested the ability of *w*Mel-HS males reared under different temperature conditions to induce cytoplasmic incompatibility (Figure 1A,B). When larvae were reared at 26 °C, *w*Mel and *w*AlbB males induced complete cytoplasmic incompatibility with uninfected females; no eggs hatched over two gonotrophic cycles. In contrast, some females that mated with *w*Mel-HS males produced a low proportion of viable offspring, indicating incomplete cytoplasmic incompatibility induction by *w*Mel-HS males. *w*Mel and *w*Mel-HS males induced partial cytoplasmic incompatibility when reared under heat stress, with weaker cytoplasmic incompatibility (and higher hatch proportions) under the more stressful temperature condition (Mann-Whitney U: Z = 5.545, *p* < 0.001). Egg hatch proportions from crosses with *w*Mel and *w*Mel-HS males did not differ from each other at 26–36.5 °C (Z = 0.781, *p* = 0.435) or 26–37.5 °C (Z = 1.419, *p* = 0.156), indicating a lack of response to selection. Hatch proportions also did not differ between gonotrophic cycles 1 and 2 for *w*Mel (Z = 0.501, *p* = 0.617) or *w*Mel-HS (Z = 0.123, *p* = 0.904) when males were reared under heat stress. In contrast to *w*Mel and *w*Mel-HS, *w*AlbB males induced complete cytoplasmic incompatibility under all rearing conditions tested, with no eggs hatching in either gonotrophic cycle.

We tested the ability of *w*Mel-HS females reared under different temperature conditions to restore compatibility in crosses with infected males (Figure 1C,D). Hatch proportions of infected females reared at 26 °C were consistently high, and did not differ between the *w*Mel, *w*Mel-HS, and *w*AlbB lines (Kruskal-Wallis: χ^2^ = 5.533, df = 2, *p* = 0.063). Egg hatch proportions declined when females were reared under heat stress conditions for the *w*Mel (Mann-Whitney U: Z = 7.416, *p* < 0.001) and *w*Mel-HS lines (Z = 7.720, *p* < 0.001) and to a lesser extent the *w*AlbB line (Z = 5.004, *p* < 0.001). The loss of compatibility was particularly severe at 26–37.5 °C where median hatch rates were below 4% for the *w*Mel and *w*Mel-HS lines. Egg hatch proportions did not differ between the *w*Mel and *w*Mel-HS lines at 26–36.5 °C (Z = 1.016, *p* = 0.308) or 26–37.5 °C (Z = 0.243, *p* = 0.810), indicating a lack of selection response. Egg hatch proportions did not increase for heat-stressed *w*Mel (Z = 0.401, *p* = 0.689) or *w*Mel-HS (Z = 0.422, *p* = 0.674) females in the second gonotrophic cycle, but did increase for *w*AlbB (Z = 2.131, *p* = 0.033), suggesting that *Wolbachia* strains can differ in their ability to recover from heat stress.

### 3.3. Response of Laboratory and Field wMel Infections to Cyclical Heat Stress

**Figure 2:**
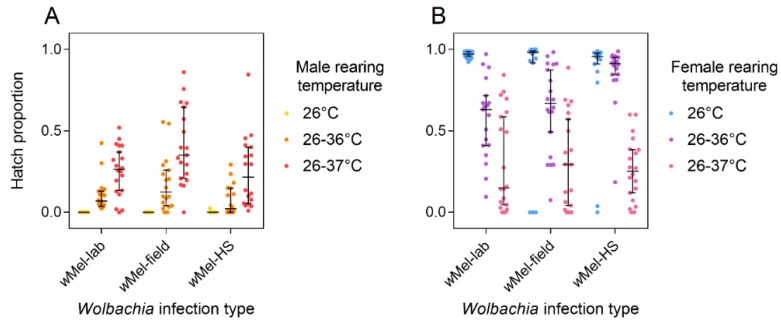
The ability of *w*Mel to induce cytoplasmic incompatibility and restore compatibility under heat stress following field deployment and selection. Males (**A**) and females (**B**) from the *w*Mel-lab, *w*Mel-field, and *w*Mel-HS (following eight generations of selection) lines were reared at a constant 26 °C, or held in incubators that cycled diurnally between 26 °C and 36 °C or 26 °C and 37 °C; (**A**) Males reared at each temperature were crossed to uninfected females to test their ability to induce cytoplasmic incompatibility; (**B**) Females reared at each temperature were crossed to infected males reared at 26 °C to test their ability to restore compatibility. Black bars indicate median egg hatch proportions with 95% confidence intervals.

We performed a second set of experiments to compare a *w*Mel strain maintained in the laboratory for approximately 60 generations (*w*Mel-lab) to a *w*Mel strain that has been in the field for seven years (*w*Mel-field) in their response to cyclical heat stress. We also tested the *w*Mel-HS line, which had now been through eight generations of selection. When reared at 26 °C, both *w*Mel-lab and *w*Mel-field males induced complete cytoplasmic incompatibility, with no hatching eggs (Figure 2A). *w*Mel-HS males reared at 26 °C induced nearly complete cytoplasmic incompatibility, though a single female produced one viable progeny. *w*Mel-lab, *w*Mel-field, and *w*Mel-HS males did not differ significantly in their ability to induce cytoplasmic incompatibility when reared at 26–36 °C (Kruskal-Wallis: χ^2^ = 5.534, df = 2, *p* = 0.063) or 26–37 °C (χ^2^ = 4.858, df = 2, *p* = 0.088). All lines induced weaker cytoplasmic incompatibility when males were reared at 26–37 °C relative to 26–36 °C (Mann-Whitney U: Z = 5.072, *p* < 0.001).

We compared the ability of *w*Mel-lab and *w*Mel-field females to restore compatibility with infected males when reared under cyclical heat stress. *w*Mel-lab and *w*Mel-field females exhibited similar hatch proportions when reared at both 26–36 °C (Z = 0.759, *p* = 0.447) and 26–37 °C (Z = 0.135, *p* = 0.889, Figure 2B). In contrast, *w*Mel-HS females had higher hatch proportions than *w*Mel-lab females when they were reared at 26–36 °C (Z = 3.601, *p* < 0.001) though not at 26–37° C (Z = 0.095, *p* = 0.928). Perhaps the *w*Mel-HS line has evolved an increase in ability to restore compatibility with infected males under 26–36 °C although not at the temperature cycle used for selection. Hatch rates were lower overall when females were reared at 26–37 °C compared to 26–36 °C, indicating a reduced ability of females to restore compatibility at higher temperatures (Z = 6.563, *p* < 0.001).

### 3.4. Wolbachia Density

**Figure 3:**
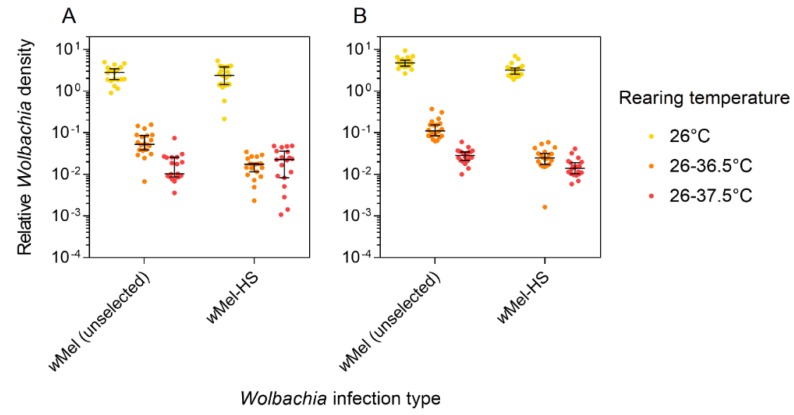
Relative *Wolbachia* density after five generations of heat stress selection. Females (**A**) and males (**B**) from the *w*Mel (unselected) and *w*Mel-HS lines were reared under three temperature conditions. Black bars indicate median densities with 95% confidence intervals.

We tested if *Wolbachia* density had changed in response to selection. After five generations of selection, we estimated *Wolbachia* density in whole *w*Mel and *w*Mel-HS adults when reared at a constant 26 °C or under two cyclical temperature conditions. These adults were taken from the same containers used for the first cytoplasmic incompatibility experiment. *Wolbachia* density was greatly reduced under heat stress in both *w*Mel (Mann-Whitney U: Z = 8.899, *p* < 0.001) and *w*Mel-HS (Z = 8.885, *p* < 0.001) lines (Figure 3). *w*Mel-HS males had lower *Wolbachia* densities than *w*Mel males at 26 °C (Z = 3.544, *p* < 0.001), 26–36.5 °C (Z = 5.396, *p* < 0.001), and 26–37.5 °C (Z = 3.746, *p* < 0.001). For females, *Wolbachia* density did not differ between the *w*Mel and *w*Mel-HS lines (all *p* > 0.05) except for when they were reared at 26–36.5 °C, where the *w*Mel line had a higher density than the *w*Mel-HS line (Z = 4.720, *p* < 0.001).

**Figure 4:**
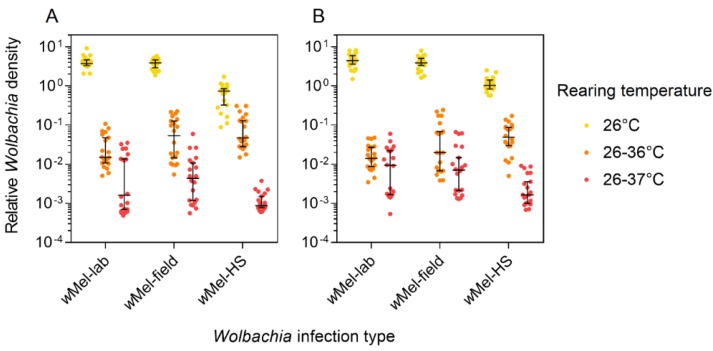
Relative *Wolbachia* density of laboratory and field-derived *w*Mel-infections. Females (**A**) and males (**B**) from the *w*Mel-lab, *w*Mel-field, and *w*Mel-HS (following eight generations of selection) lines were reared under three temperature conditions. Black bars indicate median densities with 95% confidence intervals.

We measured the *Wolbachia* density of *w*Mel-lab, *w*Mel-field, and the *w*Mel-HS line after eight generations of selection. *Wolbachia* density was greatly reduced at higher rearing temperatures for all strains (Kruskal-Wallis: df = 2, all *p* < 0.001, Figure 4). *Wolbachia* density in the *w*Mel-field and *w*Mel-lab lines did not differ at each temperature and for each sex (Mann-Whitney U: all *p* > 0.05), indicating that the infection density of *w*Mel has remained stable following field releases. In contrast, *Wolbachia* density was greatly reduced in *w*Mel-HS females (Mann-Whitney U: Z = 5.396, *p* < 0.001) and males (Z = 5.234, *p* < 0.001) at 26 °C relative to *w*Mel-lab, with *w*Mel-HS females and males having 16% and 25% the density of *w*Mel-lab females and males, respectively. *w*Mel-HS adults had a higher density than *w*Mel-lab adults when reared at 26–36 °C (Z = 4.566, *p* < 0.001) but a lower density when reared at 26–37 °C (Z = 2.839, *p* = 0.005); any effects of selection on density therefore appear to be inconsistent across conditions.

## 4. Discussion

In this study, we attempted to generate a heat-resistant strain of *w*Mel in *Ae. aegypti* that could serve as an alternative *Wolbachia* variant for disease control programs. In response to intense selection, *w*Mel did not consistently improve its thermal tolerance and only exhibited a reduced density at 26 °C compared to an unselected *w*Mel strain. We also show that *w*Mel infections from the laboratory and field did not differ in their response to heat stress or in their phenotypic effects under standard laboratory conditions, despite the infection having been maintained in field populations for seven years. These findings suggest that the *w*Mel infection is unlikely to change much in its response to heat stress after field deployment. On the other hand, our comparisons between the lab strain and field strain indicate that the infection will likely remain stable in its ability to induce cytoplasmic incompatibility under non-stressful conditions for many years following field releases.

Given that *Wolbachia* strains can differ substantially in their response to cyclical heat stress [28] and adapt rapidly to changing environments [35,37], the limited response of *w*Mel to selection for increased heat tolerance in the laboratory despite intense selection is perhaps surprising. It is possible that trait-relevant genetic variation within both the *w*Mel *Wolbachia* genome and nuclear *Aedes* genome is limited. *Drosophila* data suggest a limited ability of populations to adapt to high temperature stress, which may be related to a lack of genetic variation generally or genetic interactions with other traits [45]. Perhaps adaptation to heat stress might occur after a much longer period of selection, particularly given that females from the selected line seemed to show an increase in compatibility with infected non-stressed males after eight selection generations when the females were reared at 26–36 °C.

The comparison of field and lab strains showed no change in responses to heat stress despite lines being separated by 60 generations. In the field, the strength of selection for maintaining a high density of *Wolbachia* is unclear given that this will depend on exposure to high temperatures in undefined breeding sites. Field experiments suggest that when mosquitoes breed in Cairns during the wet season, *Wolbachia* will reduce in density sufficiently to trigger a loss of compatibility with infected males in breeding containers placed in full or partial sunlight (Ross, unpublished). However, it is not known what proportion of a mosquito population will be exposed to sufficiently high temperatures. Moreover, any costs of increased heat resistance in terms of a lower density of *Wolbachia* at 26 °C could reduce selection pressures for resistance in the case where females suffer reduced egg hatch when mated to infected males.

Our results for the field strain comparison are consistent with previous studies showing that the *w*Mel infection has remained stable in its effects after field deployment [13,14], but contrast with the several examples of *Wolbachia* infections adapting rapidly to changes in environmental conditions in other insects. There are concerns that *Wolbachia* strains in *Aedes* mosquitoes could evolve and lose effectiveness after field deployment [46]. We suspect that such evolution is unlikely in the short-term, at least for the *w*Mel infection, given that its density and ability to induce cytoplasmic incompatibility have remained stable for seven years in both the laboratory and the field.

Despite maintaining susceptibility to heat stress, the *w*Mel infection has established and persisted at a high frequency in multiple locations in Cairns, Australia [2,13], but this does not ensure the success of releases in other areas where maximum daily temperatures during the hottest times of year are higher than in release zones in Australia. Since weather reports are not necessarily good indicators of microclimates in mosquito larval habitats [47], careful monitoring will be required to determine if heat stress will be a concern for *w*Mel release programs in other areas. The deployment of alternative strains like *w*AlbB that are more thermally robust may be required in these areas for efficient population transformation and viral suppression.

## Figures and Tables

**Table 1:** Average maximum temperatures and egg hatch rates of *w*Mel-infected *Ae. aegypti* females for the first eight generations during the selection experiment. Females reared at the specified temperature were crossed to *w*Mel-infected males reared at 26 °C. Generation 3 is a relaxed generation. Only eggs and egg hatch proportions from cages that contributed to the next generation are shown. Egg hatch rates were not recorded after the eighth generation.

Generation	1	2	3	4	5	6	7	8
Mean maximum daily temperature	37.2 °C	37.3 °C	26 °C	36.9 °C	36.9 °C	37.0 °C	36.8 °C	36.8 °C
Egg hatch (*w*Mel-HS ♀ × *w*Mel 26 °C ♂)	30.02%	2.64%	69.88%	2.12%	2.87%	2.90%	3.67%	0%
Eggs scored	991	4040	934	2113	2115	8396	4741	4183

## Supplementary Materials

The following are available online at http://www.mdpi.com/2075-4450/9/3/78/s1, Figure S1: Temperatures experienced in larval rearing trays during the (A) first and (B) second cytoplasmic incompatibility experiments on *Ae*. *aegypti*, Figure S2: Proportion of eggs hatching from crosses between *w*Mel-infected *Ae. aegypti* females reared under heat stress and *w*Mel-infected males reared at 26°C during the selection experiment, Figure S3: Hatch proportions of eggs from *w*Mel-infected female *Ae*. *aegypti* reared under cyclical heat stress and held at 26°C for 1, 4 or 7 days before being crossed to *w*Mel-infected males reared at 26°C, Figure S4: *Wolbachia* density of *w*Mel, *w*MelPop and *w*AlbB strains when *Ae. aegypti* are reared under heat stress and provided with different levels of larval nutrition.
