# Supplementary figures and images for: Continued Susceptibility of the wMel Wolbachia Infection in Aedes aegypti to Heat Stress Following Field Deployment and Selection
Source: Insects. 2018 Jun 30;9(3):78. doi: 10.3390/insects9030078 (PMC6165456; doi:10.3390/insects9030078)

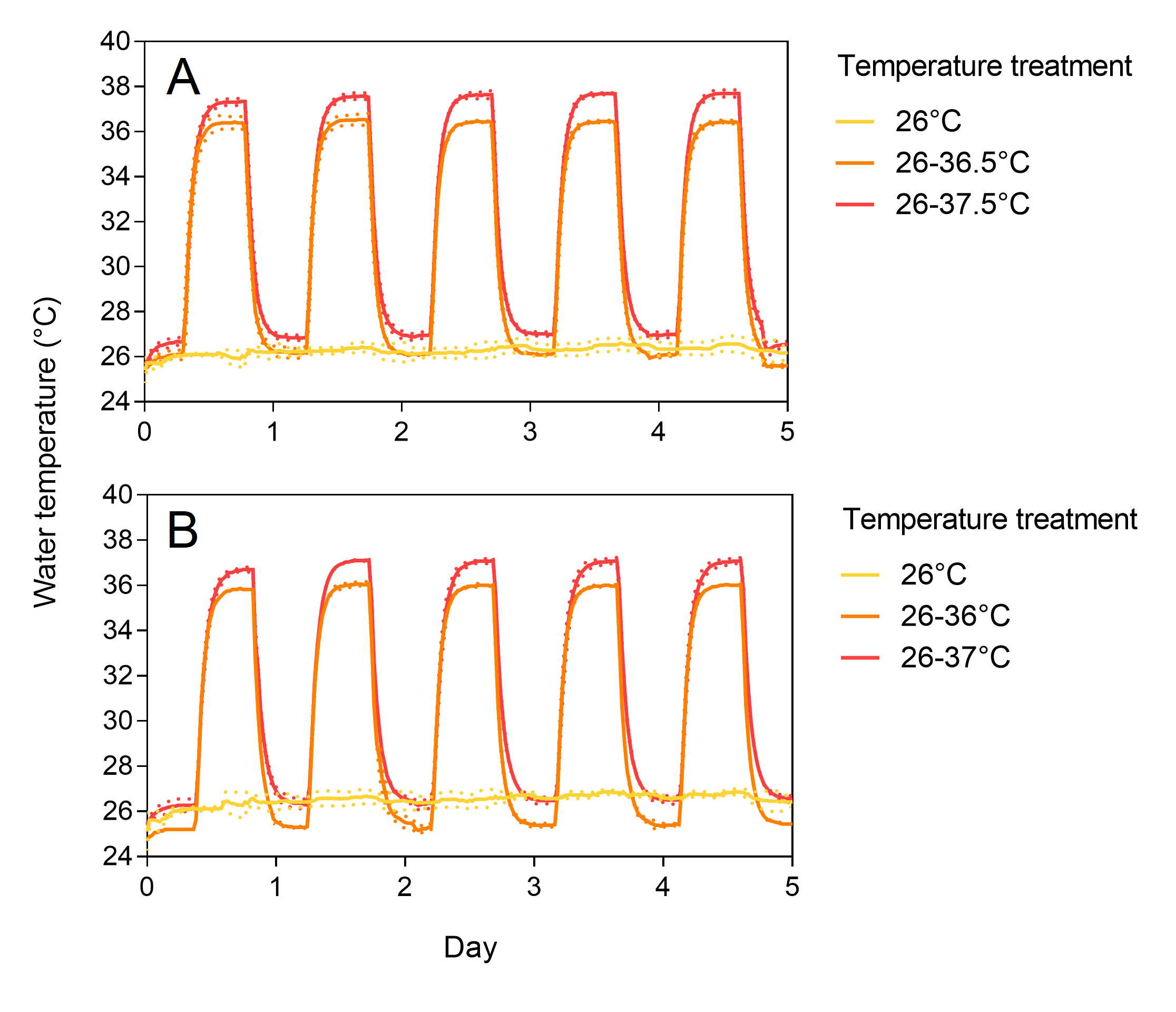

Supplement: Supplementary file 1 [file insects-09-00078-s001.zip › Figure S1.tif]

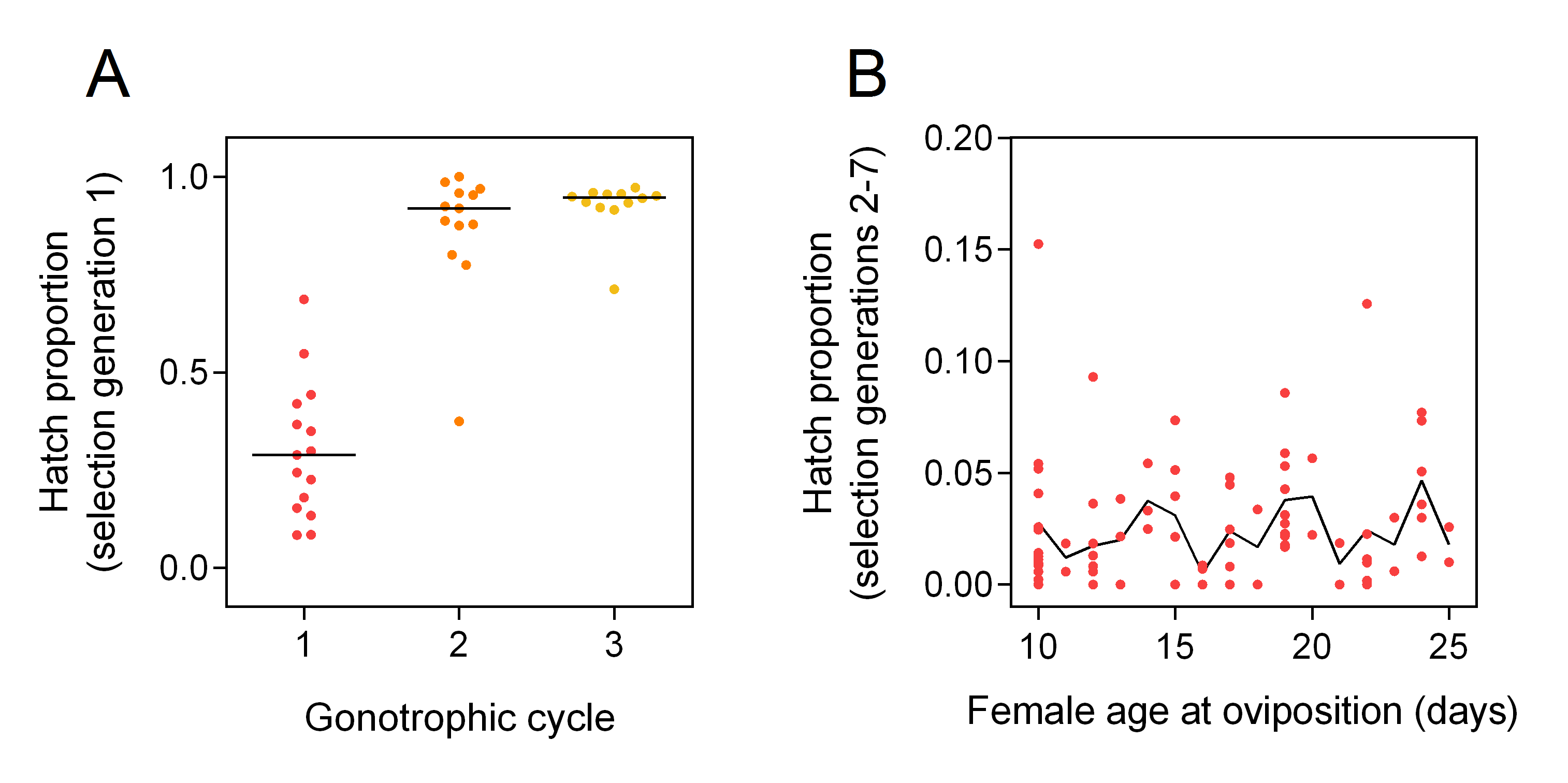

Supplement: Supplementary file 1 [file insects-09-00078-s001.zip › Figure S2.tif]

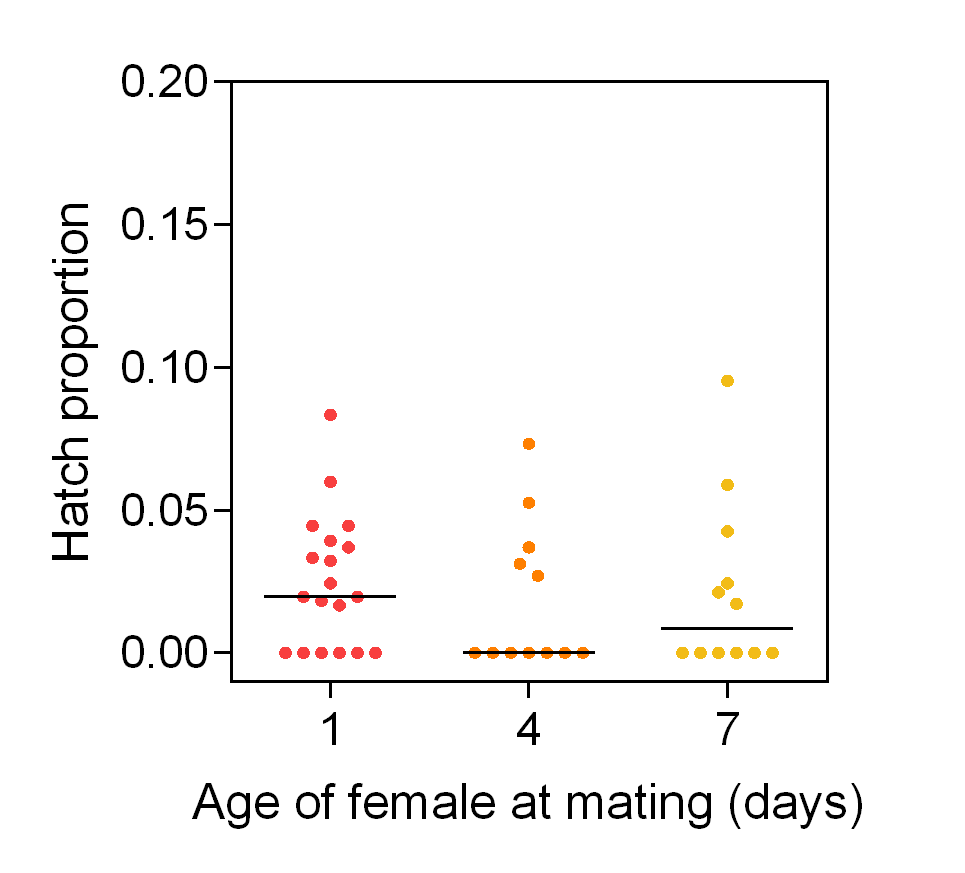

Supplement: Supplementary file 1 [file insects-09-00078-s001.zip › Figure S3.tif]

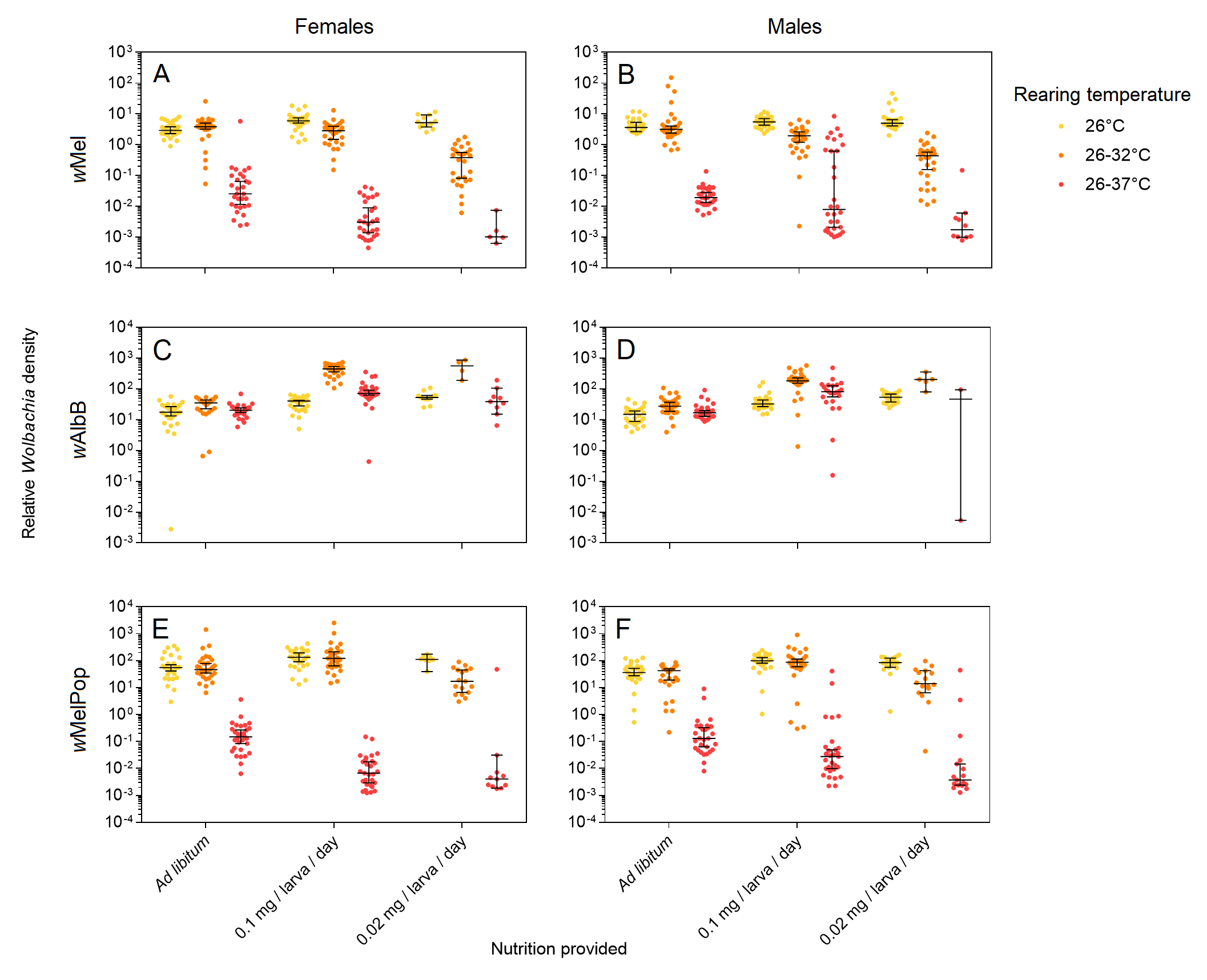

Supplement: Supplementary file 1 [file insects-09-00078-s001.zip › Figure S4.tif]
